# “I fear those things”: non-uptake of contraceptives, and barriers to use among adolescent girls and young women at high risk of HIV infection in Kampala, Uganda

**DOI:** 10.3389/frph.2023.1198672

**Published:** 2023-08-15

**Authors:** Rachel Kawuma, Jane Frances Lunkuse, Wilber Ssembajjwe, Ivy Kayesu, Matt A. Price, Debbie B. Brickley, Andrew Abaasa, Yunia Mayanja

**Affiliations:** ^1^ Medical Research Council, Uganda Virus Research Institute and London School of Hygiene and Tropical Medicine, Uganda Research Unit, Entebbe, Uganda; ^2^ Department of Epidemiology and Biostatistics, University of California, San Francisco (UCSF), San Francisco, CA, United States; ^3^ IAVI, New York, NY, United States; ^4^ Institute for Global Health Sciences, University of California, San Francisco (UCSF), San Francisco, CA, United States; ^5^ Department of Infectious Disease Epidemiology, London School of Hygiene & Tropical Medicine, London, United Kingdom

**Keywords:** adolescent girls and young women, sexual and reproductive health, contraception, utilization, non-uptake, Sub-Saharan Africa

## Abstract

**Background:**

Adolescent girls and young women involved in risky behaviors are vulnerable to multiple health problems, yet sexual and reproductive health services remain underutilized. We evaluated factors associated with non-uptake of contraceptives and barriers to use among adolescent girls and young women (14–24 years old) at high risk of HIV infection in an environment where contraceptives were provided at no cost.

**Methods:**

We conducted a mixed methods study, utilizing data from a baseline cross sectional survey and qualitative in-depth interviews. Survey participants tested negative for pregnancy and reported willingness to use contraception. Non-uptake of contraceptives was defined as not taking contraception at any study visit (baseline and throughout the study). Logistic regression model was used to assess factors associated with non-uptake of contraceptives. We purposively selected participants for interviews to discuss their knowledge and experiences with contraceptives and make suggestions to improve uptake. Qualitative data were analyzed thematically.

**Results:**

All 285 participants were included in the analysis. Out of the 285 participants 127 were not using contraceptives and of the 127, 44 (34.6%) did not take up any method throughout the study while 43 of the 83 remaining participants (who took up a method) chose male condoms only. Non-uptake of contraceptives was less likely among older women (20–24 years) (aOR = 0.32, 95% CI 0.16–0.89) compared to younger women (less than 20 years). Qualitative data showed that concerns about future fertility, fear of associated side effects and influence from close relations contributed to non-uptake of contraception.

**Conclusion:**

Non-uptake of contraceptives was common despite the promotion and provision of contraceptives in the context of a research study mainly because adolescents lack autonomy while making contraceptive decisions. Identifying and addressing their concerns and continued counselling on contraceptive use alongside condom promotion may improve uptake and utilization of contraceptives.

## Introduction

Adolescent girls and young women (AGYW). are considered a vulnerable population for HIV, contributing a substantial proportion of new HIV infections. For instance, six out of seven new HIV infections among adolescents aged 15–19 years in Sub-Saharan Africa (SSA) are among girls, with girls and young women aged 15–24 years twice as likely to be living with HIV than young men. This could be attributed to their early sexual debut compared to boys of their age, engaging in multiple sexual relationships, often times with older men and involvement in transactional sex (1, 2) Besides HIV infection, such risky sexual behavior may result in unintended pregnancies which can lead to unsafe abortions or even death if contraception is not used (3). Indeed, at least 10 million unintended pregnancies occur each year among adolescent girls aged 15–19 years in the SSA region (4), with a 54.6% prevalence in East Africa (5) and 25% in Uganda (6). The incidence of unwanted pregnancy reported in the current study was 24 out of every 100 AGYW (7). Such poor sexual and reproductive health (SRH) outcomes derail the progress of sustainable development goal 3.7 which sets out to ensure that there is “universal access to sexual and reproductive health-care services, including for family planning, information and education, and the integration of reproductive health into national strategies and programmes by 2030” (8).

Globally, the use of modern contraceptives has generally increased over the years, but it is still low in SSA (9–11) including Uganda with a prevalence of 26.6% (6). Utilization of contraceptives is lower among AGYW compared to older women aged 20–49 years old (12). This could be attributed to individual factors such as age, household/community factors which include their relationships with peers, partners and parents as well as structural factors like the long distance to a health facility (13).

While studies have assessed the need for contraception among women at high risk of HIV infection (14–16), most have been conducted among older women. Yet AGYW at risk of HIV infection may have specific challenges seeking appropriate contraception, such as socio-cultural norms against young unmarried girls being sexually active and poor attitudes from the health care workers towards young girls seeking contraception (17). It is therefore important to identify and understand the factors which influence decisions to use contraceptives and address specific barriers to their uptake among this population, which is the focus of this paper.

We explored non-uptake of contraceptives in an environment where they were provided at no cost to participants in the “*Interventions for HIV Prevention among Adolescent Girls and Young Women (IPAD)*” study, which assessed knowledge and preferences of biomedical HIV prevention methods and uptake of oral PrEP among AGYW at high risk of HIV infection from January 2019 to December 2020 (18). Reliable contraceptive methods (both hormonal and non-hormonal) including oral pills, injectables, implants and condoms were provided and promoted for use at no cost throughout the study. Therefore, the objective of this paper is to assess the factors associated with non-uptake, and barriers to use of contraceptives among AGYW at high risk of HIV in Kampala, Uganda.

## Methods

### Study design and setting

We conducted a mixed methods analysis of quantitative and qualitative data collected during the IPAD study for a year at the Good Health for Women Project (GHWP) clinic in Kampala, the capital city of Uganda. The GHWP clinic was established in 2008 to study epidemiology of HIV and STIs among women at risk of HIV infection (19), and the IPAD study represents a subset of these clinic attendees, 14–24 years old, enrolled as study participants (18). Until December 2020 when the clinic was closed, the clinic offered HIV prevention, care, and treatment services such as pre- and post-exposure prophylaxis and risk reduction counselling, SRH services such as management of STIs and provision of contraceptives to both HIV-positive and negative women and their male regular partners. GHWP and IPAD participants (and their male partners) were offered options for contraception that included both hormonal and non-hormonal methods every 3 months at clinic visits. Study follow up lasted one year.

### Study population

The IPAD study team field mobilisers recruited AGYW from urban slums in neighboring communities of the study clinic and a total of 561 potential participants were approached. Many engaged in sex work or worked in the leisure and hospitality industry. Screening into the IPAD study was done by trained study nurses and 154 were screened out. Those who were aged 14–24 years, HIV-negative, sexually active, and reported willingness to use effective contraception and go through all the study procedures were recruited. However, 93 eligible volunteers were not recruited as the final sample size of 285 was reached. The study recruited minors (14–17 years) if they were identified as emancipated or mature and were able to provide their own informed consent to participate according to national guidelines (20). Recruitment, eligibility and screening into the IPAD study has been previously described (18).

Twenty AGYW were purposively selected to participate in the qualitative component of the study. To reduce selection bias, participants of varied characteristics were included namely, hormonal contraceptive users (capsule or injectable), non-hormonal users (male condoms) and those not using any method at baseline. Other selection criteria were age (equal number of 14–19 years and 20–24 years) and level of education (none/primary or secondary).

### Data collection

Quantitative data: All participants completed an interviewer-administered questionnaire on socio-demographic and sexual behavior characteristics at each study visit every three months. In addition, participants received SRH information, HIV testing, STI screening and free condom supplies at those visits. Contraception was discussed and made available at all visits.

Qualitative data: One in-depth interview (IDI) was conducted with each participant selected into the qualitative study within one month of enrolment into the cohort to explore their knowledge and experiences with contraceptives, specifically focusing on the following themes and ideas: “factors influencing choice”, “benefits of one method compared to other”, “barriers to contraceptive use” and “suggestions to improve contraceptive uptake among young women”. The IDI was conducted in Luganda, the local language, and audio recorded.

### Study outcome

The primary outcome variable, non-uptake of contraception, was defined as a participant who was not using a hormonal or barrier contraceptive method at baseline and did not take up any of the methods that were offered or mention using a condom as a contraceptive at subsequent visits throughout the study. Non-uptake was recorded as a binary variable (yes/no) where “no” was defined as no uptake of contraception at any visit.

Independent variables were assessed at baseline and included age, education level, marital status, alcohol use, number of children, occupation, and level of income. We also collected data on alcohol use in the past one month (yes/no). Alcohol use was further assessed using a standardized WHO Alcohol Use Disorders Identification Test (AUDIT) (21) and classified into two categories i.e., low to moderate-risk drinkers (score 0–15), and high-risk drinkers/harmful or dependent (score ≥16).

### Data analysis

Quantitative data analysis: Data were double entered in Open Clinica 3.5 (Waltham, MA) cleaned, and exported to Stata 17.0 (Stata Corp, College Station, Texas, USA) for analysis. We resolved discrepancies by checking the source documents for clarification. Categorical demographic characteristics were summarized using frequencies and proportions. Continuous variables were summarized using means (SD) or medians (IQR) based on the nature of the distribution of the variables as appropriate, and standard deviations or interquartile ranges, respectively. Participant characteristics are shown overall and stratified across contraceptive uptake and compared using Chi–square test.

We fitted both Univariable and multivariable logit models. Factors considered included age, level of education, marital status, number of biological children, number of sexual partners, age of first sex, drinking of alcohol, alcohol audit, use of drugs, main job done and levels of income. After a univariable analysis, we considered factors for inclusion into a multivariable logit model basing on prior knowledge of their association with the outcome. Factors were retained in the final multivariable logistic regression model, if the likelihood ratio test *p*-value for inclusion of a factor was <0.05.

Qualitative data analysis: Data were analyzed using a framework analysis approach (22). Two researchers each read the interview transcripts to identify common and recurring codes that were linked to the research topics. Transcripts were coded manually. To increase inter-rater reliability and validity, emerging codes with similar or closer meaning were combined to create themes. Data were categorized according to themes and charted in an Excel matrix table to identify patterns and link the data to the main topics.

## Results

### Participant baseline characteristics

Out of 561 potential participants who were approached and screened, two hundred and eighty-five AGYW were enrolled and included in the analysis, representing the entire IPAD study population. The mean age at baseline was 20 years (SD ± 2.2), and 175 participants (61.4%) were aged >20 years old. More than half (54.7%) had studied up to secondary level, and 57.2% were single (never married) yet 20.3% had >10 sexual partners in the previous 3 months. One hundred and eighty-two (63.9%) participants had at least one child, the majority (80%) first had sex between 9 and 17 years of age, 148 (51.9%) took alcohol in the last one month, 84 (29.5%) worked in the hospitality industry and 174 (61.0%) earned ≤10 $ every week (Table 1). Majority of the participants (*n* = 158, 55.4%) reported use of contraception at baseline.

**Table 1:** Baseline socio-demographic characteristics of adolescent girls and young women in the IPAD study in Kampala, Uganda (Jan-October 2019), *N* = 285.

Variable	Category	(*n* = 285)	(%)
Age at baseline	14–19 years	110	38.60
	20–24 years	175	61.40
Education	None/primary	129	45.3
	Secondary+	156	54.7
Marital status	Married	84	29.5
	Separated/divorced	38	13.3
	Single/never married	163	57.2
Number of biological children	None	103	36.1
	At least one	182	63.9
Number of sexual partners in the last 3 months	<10	230	80.7
	10+	55	20.3
Age at first sexual encounter	9–17 years	228	80.0
	18–20 years	57	20.0
Alcohol use in the last one month	None	137	48.1
	Took alcohol.	148	51.9
Alcohol use (AUDIT Tool)	Low to moderate risk	244	85.6
	High risk	41	14.4
Drugs used last month	None	241	84.6
	Used drugs	44	15.4
Main Job	Sex work	60	21.1
	Hospitality	84	29.5
	No job	68	23.8
	Other	73	25.6
Average weekly income	≤10 $	174	61.0
	>10 $	111	39.0

### Proportion of non-uptake of contraceptives

**Figure 1:**
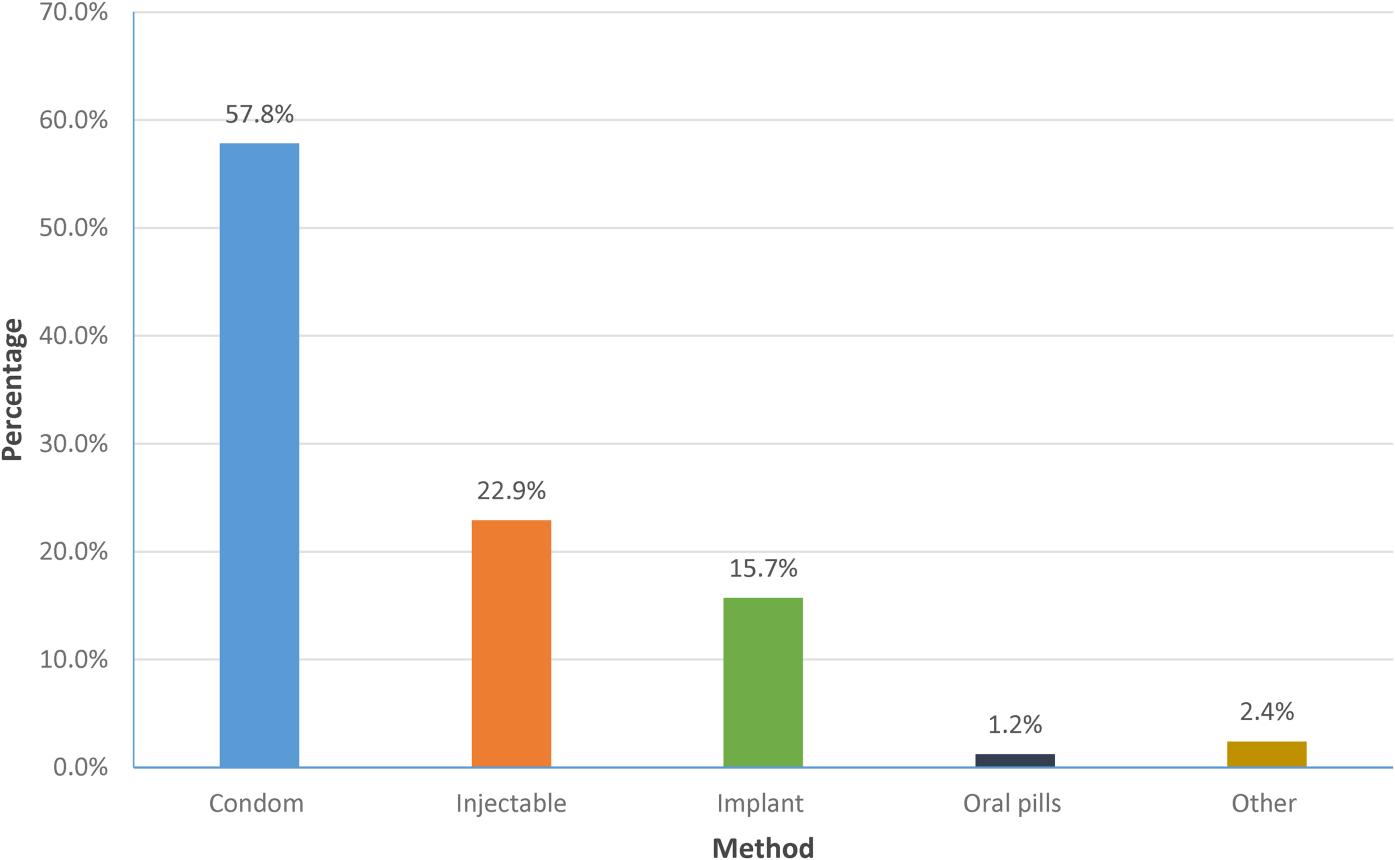
Contraceptive methods taken up after baseline among 83 adolescent girls and young women not on contraception in Kampala, Uganda (Jan 2019-December 2020).

One hundred and twenty-seven (44.6%) AGYW were not using contraceptives or condoms at baseline, of whom 44 (34.6%, or 15.4% of the entire study population) did not take up a method throughout the study, despite all participants reporting a willingness to consider contraception (Table 2). However, of the 83 (out of 127) who took up a method during the study, 43 (57.8%) took up only male condoms (Figure 1).

**Table 2:** Non-uptake of contraceptives despite free provision among 127 adolescent girls and young women not already on contraception in Kampala, Uganda (Jan-October 2019).

Variable	Category	Overall *N* = 127	(%)	Non-uptake *N* = 44	(%)	Took up *N* = 83	(%)	*P*-valve
Age at baseline	14–19 years	64	50.4	27	61.4	37	44.6	0.072
	20–24 years	63	49.6	17	38.6	46	55.4	
Education	None/primary	50	39.4	17	38.6	33	40.0	0.902
	Secondary+	77	60.6	27	61.4	50	60.0	
Marital status	Married	31	24.4	12	27.3	19	23.0	0.807
	Separated/divorced	19	15.0	7	15.9	12	14.0	
	Single/never married	77	60.6	25	56.8	52	63.0	
Number of biological children	None	62	48.8	25	56.8	37	44.6	0.189
	At least one	65	51.2	19	43.2	46	55.4	
Number of sexual partners in the last 3 months	<10	112	88.2	39	88.6	73	88.0	0.909
	10+	15	11.8	5	11.4	10	12.0	
Age at first sexual encounter	9–17 years	99	78.0	39	88.6	60	72.3	0.034
	18–20 years	28	22.0	5	11.4	23	27.7	
Alcohol in the last one month	None	73	57.5	27	61.4	46	55.4	0.519
	Took alcohol	54	42.5	17	38.6	37	44.6	
Alcohol use (AUDIT Tool)	Low to moderate	117	92.1	42	95.5	75	90.4	0.311
	High risk	10	07.9	2	04.5	8	09.6	
Drugs used last month	None	109	85.8	40	90.9	69	83.1	0.232
	Used drugs	18	14.2	4	09.1	14	16.9	
Main job	Sex work	25	19.7	7	15.9	18	21.7	0.648
	Hospitality	45	35.4	18	40.9	27	32.5	
	No job	28	22.1	8	18.2	20	24.1	
	Other	29	22.8	11	25.0	18	21.7	
Average weekly income	= <10 $	85	66.9	29	65.9	56	67.5	0.859
	>10 $	42	33.1	15	34.1	27	32.5	

### Factors associated with non-uptake of contraceptives

In the unadjusted analysis, age at baseline, level of education and being screened as high-risk drinkers were associated with non-uptake of contraception. Participants who were >20 years old, those who attained secondary level and above and those who were screened as high-risk alcohol drinkers were more likely to not take up contraceptives throughout the study period (Table 3).

**Table 3:** Factors associated with non-uptake of contraceptives among 285 adolescent girls and young women in Kampala, Uganda (Jan 2019-December 2020).

Variable	Category	uOR (95% CI)	*p*-value^a^	aOR (95% CI)	*p*-value^a^
Age at baseline	14–19years	1	0.009	1	0.024
	20–24 years	0.33 (0.17–0.64)		0.32 (0.16–0.63)	
Education	None/primary	1	0.334	1	0.488
	Secondary+	1.38 (0.71–2.66)		1.49 (0.76–2.94)	
Marital status	Married	1	0.845		
	Separated/divorced	1.35 (0.49–3.77)			
	Single/never married	1.09 (0.51–2.28)			
Number of biological children	None	1	0.002		
	At least one	0.36 (0.19–0.69)			
Number of sexual partners in the last 3 months	<10	1	0.126		
	10+	0.49 (0.18–1.31)			
Age at first sexual encounter	9–17 years	1	0.099	** **	** **
	18–20 years	0.47 (0.17–1.24)			
Alcohol use in the last one month	None	1	0.054		
	Took alcohol	0.53 (0.27–1.02)			
Alcohol use (AUDIT Tool)	Low to moderate risk	1	**0**.**023**		
	High risk	0.25 (0.05–1.06)		0.26 (0.06–1.14)	0.07
Drugs used last month	None	1	0.180		
	Used drugs	0.50 (0.17–1.48)			
Main job	Sex work	1	0.312		
	Hospitality	2.06 (0.80–5.31)			
	No job	1.01 (0.34–2.97)			
	Other	1.34 (0.49–3.71)			
Average weekly income	= <10 $	1	0.047		
	>10 $	0.78 (0.39–1.53)			

uOR, unadjusted odds ratio; aOR, adjusted odds ratio; CI, confidence interval.

^a^ Likelihood ratio test.

In the adjusted analysis, non-uptake of contraceptives was less likely among older women 20–24 years (aOR = 0.32, 95% CI 0.16–0.63). Reported high risk alcohol use was borderline associated with non-uptake (aOR = 0.26, 95% CI 0.06–1.14). Education was not associated with non-uptake in the adjusted analysis.

## Qualitative results

### Characteristics of participants

Twenty out of the 285 AGYW participated in the qualitative component of the study. They included 11 participants who were using hormonal contraception (implant and injectable), 6 on non-hormonal contraception (male condoms) and two who were not using any contraceptive method. Half of these were aged ≥20 years. Thirteen had attained at least a secondary education, 5 had a primary education and 2 did not have any form of education. Most of them (14) lived with relatives such as uncles, aunts, and older siblings, 3 with friends and another 3 with either a boyfriend or husband.

Participants broadly discussed what they perceived as barriers to contraceptive use, some of which they had not personally experienced but either knew someone who had gone through them or had heard about them. Several themes were identified including concerns about future fertility, fear of related side effects and influence from close relations as explained below.

### Concerns about future fertility

Misconceptions and misinformation held about the hormonal methods discouraged some from taking them up. “*Some people say that they bring cancer; others say that they make you bleed until the drug gets finished*”. 20- year-old condom user with no education.

“*There are people who say that when you swallow pills, they remain in a person's body. Another person says that you will not produce again and yet there is someone who wants to give birth again in the future*”. 22- year-old non-user with no education.

These misconceptions exacerbated the fear that using such methods would have an impact on future fertility. “*I can't use those other family planning methods; I haven't yet given birth and I hope to give birth. What if I reach the time of giving birth when my ovules got burnt?*” 20- year-old condom user with primary education.

### Related side effects

Most of the participants discussed experiences of close relations who experienced side effects because of using contraceptives which in turn could affect their perceptions of the methods. For instance, a 17-year-old non-user with secondary education narrated that, “*my mother bled a lot, and she was as if she had lost a baby, but what caused all of that was family planning tablets, so I feared.*”

Another one stated that, “*I have a friend who has used the injection and it made her bleed for almost 3 months, except on few occasions when she would go to the hospital and get some medication and stop for like 3 days and then resume again. So that one cannot use it again*”. 19-year-old hormonal method user, with a secondary education.

Reports included misperceptions and exaggerations about side effects. One participant likened the side effects of contraceptives to HIV/AIDS-related symptoms, saying, “*There are some people, to whom those things can make sick and they lose weight, and then people can tell immediately that she is using family planning, and some people can think that it is HIV/AIDS and yet it is an effect of family planning*.” 19-year-old non-user with a primary education.

### Influence from close relations

Close relations such as parents sometimes discouraged these young women from using contraceptives. For example, a 17-year-old condom-user with secondary education disclosed that her mother didn't want her to use contraceptives, saying that, “*I fear those things, other people even say that when they inject you, if you haven't given birth yet, your eggs can die. So, I can't use them. And also, my mother doesn't want me to use them.*”

Some participants were dissuaded by their partners, such as this one who had this to say about her partner's attitude: “you *also know how boys are; he is young, he can get another girl that side where he works from, he already rents and lives on his own…and he doesn*’*t want me to use contraceptives*.” 20-year-old hormonal method-user with secondary education.

## Discussion

This study found that one out of every six AGYW participating in the IPAD study did not take up any contraceptive method despite receiving education to use them and the contraceptives being freely provided in the study clinic. While previous studies mostly attributed non-uptake of contraceptives to not being able to access them (23, 24), in this study both hormonal and non-hormonal methods were provided; yet we still report low uptake in this cohort of sexually active AGYW who report behavior that puts them at risk for unintended pregnancy and HIV. Therefore, as suggested by Harrington EK., et al. and Ahinkorah BO., et al. (13, 16), addressing the unmet need for contraception alone may not improve SRH outcomes. It may be equally important to address the values and preferences held by the users which may influence decisions either to take or not take up any contraceptive method.

Like in other studies (25), we found that those who were older (20–24 years) were more likely to use contraceptives. This could be because they may be in more stable relationships, possibly married and have had children compared to the younger ones (14–19 years) who have not yet had children and want to ensure future reproductive opportunities are preserved (9). Being able to maintain one's fertility to have children later was emphasized in their narratives, and in the African setting, having children proves that one is fertile, and motherhood is typically highly desired (26). Indeed, in some settings, use of contraceptives especially among young and unmarried women is discouraged, and some have been labelled as “spoilt” for choosing to use them (16, 27).

Our participants had concerns regarding the use of hormonal methods which they believed to cause infertility, as has been documented in many studies (28–30). This is largely attributed to misconceptions they had about the possible side effects caused by these methods. For instance, in our study like in others (16, 26), some young women mentioned that using contraception may “spoil their eggs (ova)” thus making them unable to bear children. Others discussed examples of peers who had used the injectable, and it took them some time after they had gone off the injection to get pregnant. Indeed, depo medroxyprogesterone + acetate (DMPA) causes delayed return to fertility even after stopping its use (31). Studies have related these misconceptions to the lack of sufficient knowledge about how hormonal methods work in one's body (16, 27). Therefore, health programs need to provide culturally relevant information about contraceptive use while addressing misconceptions and providing on-going counselling for side effects which occur after contraceptives are stopped.

A high proportion of the AGYW in this study reported using male condoms as their contraceptive method. Preference to use condoms has been noted in other studies (27, 32). This could be because condoms are easily accessed, cheap and probably the most promoted method of family planning after abstinence among young adolescents, particularly of school-going age (27, 28). Given that our study enrolled young people who perceived themselves to be at high risk of HIV infection due to their involvement with multiple sexual partners, they probably used condoms both as contraceptives but also to avoid other outcomes such as HIV and STIs. It is therefore not surprising that a high proportion of our participants used the condom, which served a dual purpose of preventing HIV/STIs and pregnancy when used correctly and consistently (33). There may be high demand for multi-purpose prevention technologies which can meet ‘multiple’ SRH needs of AGYW rather than having to rely on one method at every sexual encounter (34).

Other significant relationships, including parents and sexual partners influenced decisions to take up certain contraceptives. For instance, in our study, some felt they could not take up any method because their mothers objected. Other studies have documented the importance of parent and adolescent communication as a driver for the uptake of sexual and reproductive health services (35). In some instances, those who received more education about contraceptives from their parents used them more than those who did not (29) and similarly, those with supportive partners were more likely to use contraceptives than those who had non-supportive ones (36). These findings support ours, that the decisions regarding contraceptive uptake by our study participants are made not only at an individual level but are also influenced at relational and community levels. Tackling these different layers is important to support uptake and use in the long term.

This study had some limitations. Firstly, we did not assess for factors associated with non-uptake of contraceptives at the different study time points (every 3 months). Though uptake of contraception may have happened at subsequent study visits, our factors were measured at baseline and the qualitative interviews were conducted within one month of enrolling the participants. Therefore, some of these factors could change over time, particularly behavioral variables. Secondly, we were not able to compare the outcomes within different categories, for instance age vs. level of education. Finally, the use of male condoms as a contraceptive method could have been reported more than they were used, since the AGYW are likely to have used them for both protection against HIV and other STIs. Despite these limitations, our mixed methods approach, using both quantitative and qualitative methods to explore non-uptake of contraceptives when products are available, while sharing experiences and concerns that AGYW had with contraceptives contributes to the growing literature on issues around non-uptake of contraception. The findings highlight the need for more multi-purpose prevention products which allow the user an opportunity to choose a convenient method that can be used for varied reasons. As such, it prevents monotony and the different shortcomings that may be associated with using one preventive method over the other, while at the same time allowing one to have control over a method of their choice.

## Conclusion

The reasons for non-uptake of contraceptives among this group were similar to those documented among other populations. We observed that the lack of autonomy due to age, as well as involvement in risky sexual behaviors, often with multiple sexual partners increases their vulnerability to unintended pregnancies, STIs, and HIV. It is important that their expressed barriers are considered and addressed. In addition, the qualitative data set pointed out the misconceptions held about modern contraceptives, particularly the likelihood that they cause infertility, a belief that is held amongst those who desire to have children.

Therefore, understanding AGYW's reasons for non-uptake of contraceptives, their preferences for certain methods over others is important. For instance, their preference for condoms as opposed to long-acting contraceptive methods may be pointing to the need to invest in the development of multi-purpose prevention products and promote their use. Secondly, while designing interventions, it is important to not only focus on the individual but also consider other layers such as the relational, environmental and policy factors that may influence AGYW decisions to either take up a contraceptive method or not. Finally, combating misconceptions is challenging. Education and information must remain a central element of any program to provide contraception, and this education should start early, among school age girls. Considering all these elements could increase uptake and use of family planning services and improve the sexual reproductive health outcomes among this population.

## Data Availability

The original contributions presented in the study are included in the article/Supplementary Material, further inquiries can be directed to the corresponding author.

## References

[1] George G Beckett S Reddy T Govender K Cawood C Khanyile D Determining HIV risk for adolescent girls and young women (AGYW) in relationships with “Blessers” and age-disparate partners: a cross-sectional survey in four districts in South Africa. BMC Public Health. (2022) 22(1):1–8. 10.1186/s12889-022-13394-4 35568839 PMC9107706

[2] Arije O Udoh E Ijadunola K Afolabi O Aransiola J Omoregie G Combination prevention package of interventions for reducing vulnerability to HIV among adolescent girls and young women in Nigeria: an action research. PLoS One. (2023) 18(1):e0279077. 10.1371/journal.pone.0279077 36652442 PMC9847984

[3] Ayalew HG Liyew AM Tessema ZT Worku MG Tesema GA Alamneh TS Prevalence and factors associated with unintended pregnancy among adolescent girls and young women in Sub-Saharan Africa, a multilevel analysis. BMC Women’s Health. (2022) 22(1):464. 10.1186/s12905-022-02048-7 36404306 PMC9677641

[4] Adolescent Pregnany. (2020). Available at: https://www.who.int/news-room/fact-sheets/detail/adolescent-pregnancy (Cited January, 2020).

[5] Worku MG Tessema ZT Teshale AB Tesema GA Yeshaw Y. Prevalence and associated factors of adolescent pregnancy (15–19 years) in east Africa: a multilevel analysis. BMC Pregnancy Childbirth. (2021) 21(1):253. 10.1186/s12884-021-03713-9 33771106 PMC7995759

[6] Uganda Demographic and Health Survey 2016. (2018). Available at: http://dhsprogram.com/pubs/pdf/FR333/FR333.pdf

[7] Namukisa M Kamacooko O Lunkuse JF Ruzagira E Price MA Mayanja Y. Incidence of unintended pregnancy and associated factors among adolescent girls and young women at risk of HIV infection in Kampala, Uganda. Front Reprod Health. (2023) 5:1089104. 10.3389/frph.2023.1089104 36910339 PMC9995850

[8] Sustainable Development Goals. (2015). Available at: https://www.un.org/sustainabledevelopment/blog/2015/12/sustainable-development-goals-kick-off-with-start-of-new-year/.

[9] Sserwanja Q Musaba MW Mukunya D. Prevalence and factors associated with modern contraceptives utilization among female adolescents in Uganda. BMC women’s Health. (2021) 21(1):1–7. 10.1186/s12905-021-01206-7 33568124 PMC7877106

[10] Ahinkorah BO Ameyaw EK Seidu AA Agbaglo E Budu E Mensah F Sexual violence and unmet need for contraception among married and cohabiting women in Sub-Saharan Africa: evidence from demographic and health surveys. PLoS One. (2020) 15(11):e0240556. 10.1371/journal.pone.0240556 33141830 PMC7608905

[11] Lunani LL Abaasa A Omosa-Manyonyi G. Prevalence and factors associated with contraceptive use among Kenyan women aged 15-49 years. AIDS Behav. (2018) 22(Suppl 1):125–30. 10.1007/s10461-018-2203-5 29943124 PMC6132050

[12] Amouzou A Jiwani SS da Silva ICM Carvajal-Aguirre L Maïga A Vaz LME. Closing the inequality gaps in reproductive, maternal, newborn and child health coverage: slow and fast progressors. BMJ Glob Health. (2020) 5(1):e002230. 10.1136/bmjgh-2019-002230 32133181 PMC7042586

[13] Ahinkorah BO Hagan JE Jr. Seidu AA Sambah F Adoboi F Schack T Female adolescents’ reproductive health decision-making capacity and contraceptive use in Sub-Saharan Africa: what does the future hold? PLoS One. (2020) 15(7):e0235601. 10.1371/journal.pone.0235601 32649697 PMC7351194

[14] Abaasa A Todd J Mayanja Y Price M Fast PE Kaleebu P Use of reliable contraceptives and its correlates among women participating in simulated HIV vaccine efficacy trials in key-populations in Uganda. Sci Rep. (2019) 9(1):15418. 10.1038/s41598-019-51879-2 31659225 PMC6817867

[15] Kusemererwa S Abaasa A Onyango M Nel AM Isaacs M Asiki G. Contraceptive preference among women at risk of HIV acquisition in a preparatory screening study for a phase III microbicide trial in South Western Uganda. AIDS Behav. (2018) 22(Suppl 1):131–8. 10.1007/s10461-018-2177-3 29855975 PMC6128163

[16] Harrington EK Casmir E Kithao P Kinuthia J John-Stewart G Drake AL “Spoiled” girls: understanding social influences on adolescent contraceptive decision-making in Kenya. PLoS One. (2021) 16(8):e0255954. 10.1371/journal.pone.0255954 34383836 PMC8360567

[17] Jonas K Duby Z Maruping K Harries J Mathews C. Rumours, myths, and misperceptions as barriers to contraceptive use among adolescent girls and young women in South Africa. Front Reprod Health. (2022) 4:960089. 10.3389/frph.2022.960089 36406890 PMC9673823

[18] Mayanja Y Kamacooko O Lunkuse JF Muturi-Kioi V Buzibye A Omali D Oral pre-exposure prophylaxis preference, uptake, adherence and continuation among adolescent girls and young women in Kampala, Uganda: a prospective cohort study. J Int AIDS Soc. (2022) 25(5):e25909. 10.1002/jia2.25909 35543110 PMC9092160

[19] Vandepitte J Bukenya J Weiss HA Nakubulwa S Francis SC Hughes P HIV and other sexually transmitted infections in a cohort of women involved in high-risk sexual behavior in Kampala, Uganda. Sex Transm Dis. (2011) 38(4):316–23. 10.1097/OLQ.0b013e3182099545 23330152 PMC3920055

[20] National Guidelines for Research Involving Humans as Research Participants Kampala. (2014). Available at: https://www.uncst.go.ug/guidelines-and-forms/

[21] Babor TF Higgins-Biddle JC Saunders JB Monteiro MG. The alcohol use disorders identification test. Geneva: World Health Organization Geneva (2001).

[22] Srivastava A Thomson SB. Framework analysis: a qualitative methodology for applied policy research. (2009).

[23] Teshale AB. Factors associated with unmet need for family planning in Sub-Saharan Africa: a multilevel multinomial logistic regression analysis. PLoS One. (2022) 17(2):e0263885. 10.1371/journal.pone.0263885 35143584 PMC8830726

[24] Ti A Soin K Rahman T Dam A Yeh PT. Contraceptive values and preferences of adolescents and young adults: a systematic review. Contraception. (2022) 111:22–31. 10.1016/j.contraception.2021.05.018 34077748

[25] Angdembe MR Sigdel A Paudel M Adhikari N Bajracharya KT How TC. Modern contraceptive use among young women aged 15–24 years in selected municipalities of western Nepal: results from a cross-sectional survey in 2019. BMJ open. (2022) 12(3):e054369. 10.1136/bmjopen-2021-054369 35338056 PMC8961113

[26] Sedlander E Bingenheimer JB Thiongo M Gichangi P Rimal RN Edberg M “They destroy the reproductive system”: exploring the belief that modern contraceptive use causes infertility. Stud Fam Plann. (2018) 49(4):345–65. 10.1111/sifp.12076 30411794 PMC6518934

[27] Mulubwa C Munakampe MN Namakula H Hernandez A Ssekamatte T Atuyambe LM Framing contraceptive use motivations among adolescents and young adults living in informal settlements in kira municipality, wakiso district, Uganda. Front Glob Womens Health. (2021) 2:658515. 10.3389/fgwh.2021.658515 34816215 PMC8594010

[28] Chimatiro CS Mpachika-Mfipa F Tshotetsi L Hajison PL. School-going adolescent girls’ preferences and views of family planning services in phalombe district, Malawi: a descriptive, cross-sectional study. PLoS One. (2022) 17(5):e0267603. 10.1371/journal.pone.0267603 35503775 PMC9064102

[29] Gbagbo FY. Contraceptive use among basic school pupils in Ghana: a case study of a municipality. Int J Pediatr. (2020) 2020:7521096. 10.1155/2020/7521096 33029154 PMC7532398

[30] Walker AW Stern L Cipres D Rodriguez A Alvarez J Seidman D. Do adolescent women’s contraceptive preferences predict method use and satisfaction? A survey of northern California family planning clients. J Adolesc Health. (2019) 64(5):640–7. 10.1016/j.jadohealth.2018.10.291 30612809 PMC6538030

[31] Taylor DJ Halpern V Brache V Bahamondes L Jensen JT Dorflinger LJ. Ovulation suppression following subcutaneous administration of depot medroxyprogesterone acetate. Contraception: X. (2022) 4:100073. 10.1016/j.conx.2022.100073 35281554 PMC8907671

[32] Oppong FB Logo DD Agbedra SY Adomah AA Amenyaglo S Arhin-Wiredu K Determinants of contraceptive use among sexually active unmarried adolescent girls and young women aged 15-24 years in Ghana: a nationally representative cross-sectional study. BMJ Open. (2021) 11(2):e043890. 10.1136/bmjopen-2020-043890 33550261 PMC7925931

[33] Beksinska M Wong R Smit J. Male and female condoms: their key role in pregnancy and STI/HIV prevention. Best Pract Res Clin Obstet Gynaecol. (2020) 66:55–67. 10.1016/j.bpobgyn.2019.12.001 32007451

[34] Abdool Karim S Baxter C Frohlich J Abdool Karim Q. The need for multipurpose prevention technologies in Sub-Saharan Africa. BJOG Int J Obstet Gynaecol. (2014) 121:27–34. 10.1111/1471-0528.12842 PMC4206830 25335838

[35] Kassa GM Arowojolu AO Odukogbe AA Yalew AW. Prevalence and determinants of adolescent pregnancy in Africa: a systematic review and meta-analysis. Reprod Health. (2018) 15(1):195. 10.1186/s12978-018-0640-2 30497509 PMC6267053

[36] Agyekum MW Henry EG Kushitor MK Obeng-Dwamena AD Agula C Opoku Asuming P Partner support and women’s contraceptive use: insight from urban poor communities in Accra, Ghana. BMC Women’s Health. (2022) 22(1):256. 10.1186/s12905-022-01799-7 35752803 PMC9233795

